# Individualized Experience With Percutaneous Transcatheter Closure of Multiple Atrial Septal Defects: A Single-Center Study

**DOI:** 10.3389/fcvm.2021.628322

**Published:** 2021-02-15

**Authors:** Zhenquan Wang, Yi Zhan, Jiahui Jin, Tingting Wu, Songyue Zhang, Huixian Qiu, Qiaoyu Wang, Rongzhou Wu

**Affiliations:** Children's Heart Center, The Second Affiliated Hospital and Yuying Children's Hospital, Wenzhou Medical University, Wenzhou, China

**Keywords:** atrial septal defect, transcatheter closure, device closure, complete closure, atrioventricular block

## Abstract

Multiple atrial septal defects (ASDs) are one type of secundum ASD, most of which have an atrial septal aneurysm or long interdefect distance. In our retrospective single-center study, we reviewed different closure strategies for multiple ASDs. We analyzed 50 patients who underwent percutaneous transcatheter closure from May 2011 to July 2019. Information on the patients' characteristics, operation procedure, occluder selection, and complications was collected. According to the feature of the defects and device choice, multiple ASDs were divided into five groups. A successful operation was achieved in every patient. A total of 50 patients were implanted with 58 devices, with 26 patients implanted with a single standard ASD occluder (ASDO); six patients were implanted with double standard ASDOs, and only one patient was implanted with three standard ASDOs. There were 17 patients whose closure was made using the small-waist–big-edge ASDO. Seventy-six percent of the patients (38/50) had an immediate residual shunt. During the mean follow-up of 25.76 ± 22.53 months, the complete closure rate was 92%. Except for two patients with a transient atrioventricular block, individualized experience with percutaneous transcatheter closure for multiple ASDs was effective in a single-center study. After a mid- to long-term follow-up, the multiple ASDOs and small-waist–big-edge ASDO had no serious adverse events or complications.

## Introduction

Atrial septal defects (ASDs) are a common congenital heart disease, and percutaneous transcatheter intervention is an established treatment method. However, percutaneous transcatheter intervention's effectiveness is limited when a complex structure is present, such as ASD with deficient rims, atrial septal aneurysm (ASA), and multiple ASDs with a long interdefect distance (IDDs). Multiple ASDs account for approximately 10% of the total cases. Since Hijazi reported successful closure of multiple ASDs, many closure strategies have been proposed depending on the specific ASDs (1). Farhaj et al. reported on 150 multihole secundum ASD patients who underwent treatment with different techniques and devices and achieved good results. They reported that the immediate complete closure rate was 61.3% and that the sixth-month complete closure rate was 77.2% (2). Many closure strategies and different devices have been used, but there were fewer reported with a higher complete closure rate (3).

While a single ASD occluder (ASDO) could provide partial closure of multiple ASDs, residual shunts (RSs) are common. For complete closure, we would apply multiple ASDOs, Amplatzer PFO occluder or a Cribriform Amplatzer device for multiple fenestrations or ASA. Because of a desire to avoid using multiple occluders, the small-waist–big-edge ASDs occluder (small-waist–big-edge ASDO) was designed. It is available in sizes between 6 and 24 mm, with 12-mm increments in left discs and 8-mm increments in the right, providing coverage for wider atrial septum tissue. It would be a novel approach for percutaneous transcatheter closure to use on multiple ASDs.

This retrospective study reported our single-center experience of percutaneous transcatheter closure for multiple ASDs, collecting information on the patients' characteristics, device choice, and follow-up results. This study aimed to summarize the safe, effective, individualized closure experience for multiple ASDs.

## Methods

### Patients

This study included 50 patients with multiple ASDs referred to a single medical center (The Second Affiliated Hospital and Yuying Children's Hospital, Institute of Cardiovascular Development and Translational Medicine, Wenzhou Medical University, Wenzhou, Zhejiang), between May 2011 and July 2019. All patients were diagnosed by transthoracic echocardiography (TTE) or transesophageal echocardiography (TEE) and met the criteria for percutaneous transcatheter closure. Information on the patients' characteristics included demographic data, the number of defects, size of all defects, IDD, the ASA diameter, and the size of the device used. This study was approved by the ethics committee of the Second Affiliated Hospital of Wenzhou Medical University. Informed consent was obtained from each patient's parents, legal guardian, or themselves.

### Classification of the Patients

**Figure 1:**
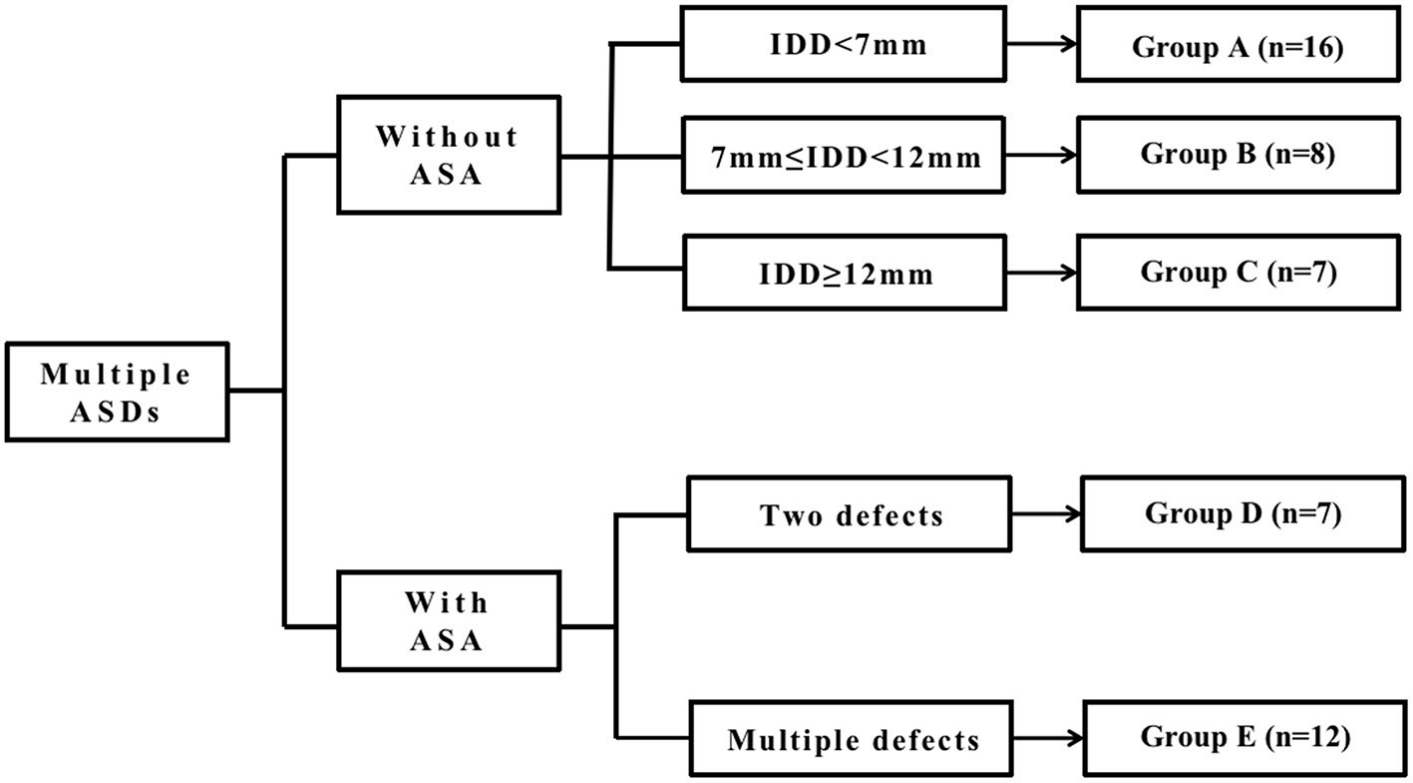
The classification standard of all patients. ASD, atrial septal defect; IDD, interdefect distance; ASA, atrial septal aneurysm.

We divided all patients into two major groups depending on whether they had ASA or not and then classified the patients without ASA into three groups: Groups A, B, and C, according to the IDD. We classified the patients with ASA into two groups, Groups D and E, according to the number of defects on the atrial septum. As the fenestrated ASDs (F-ASDs) had a closure strategy similar to ASD with ASA, we classified these patients into the ASA group. The classification standard is explained in Figure 1.

In group A, there is a large central defect (medium or small) with two or three surrounding defects. The central defect is the closed point. The distance from the farthest defect on the rim to the center defect is <7 mm.

In group B, there are two defects, and the distance between the defects is >7 mm but <12 mm.

In group C, there are two or three defects, and the center defect is the closed point. The distance from the farthest defect on the rim to the center defect is >12 mm.

Group D is considered ASDs with ASA and has two defects.

Group E is considered to be ASDs with ASA and multiple defects. As F-ASD has a similar closing strategy to ASDs with ASA, we classified these patients into this group.

The defects were classified as large (>15 mm), moderate (5–15 mm), and small (<5 mm). The IDD was defined as the distance from the central defect (implanting hole) to the surrounding hole, which needed to be covered. The ASA met the following criteria (4, 5): (1) the width of the aneurysmal base is more than 15 mm; (2) the excursion of ≥10 mm into the double atrium or the sum of bilateral excursion of >10 mm.

### Devices

**Figure 2:**
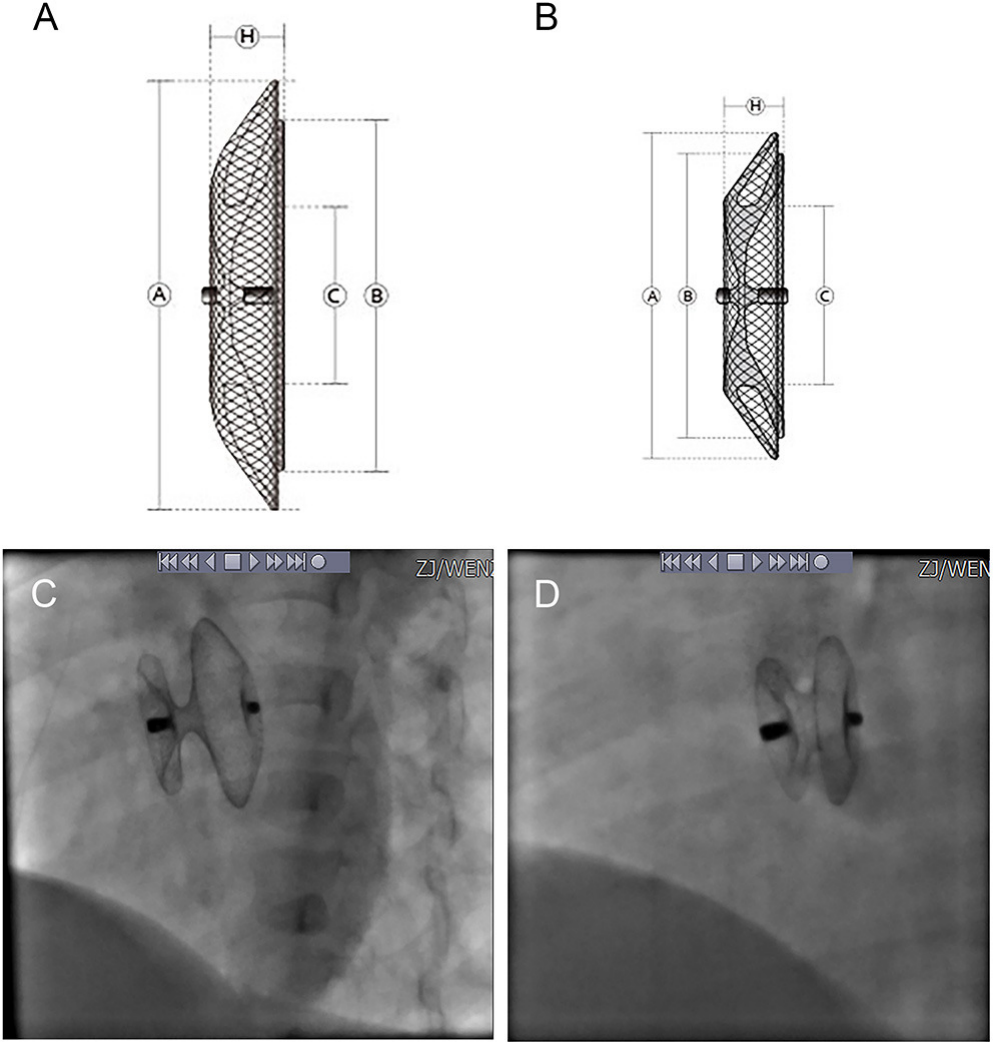
Product structure diagram and DSA image diagram of small-waist–big-edge **(A,C)** and standard **(B,D)** ASDO. ASDO, atrial septal defect occluder; ASA, atrial septal aneurysm.

**Figure 3:**
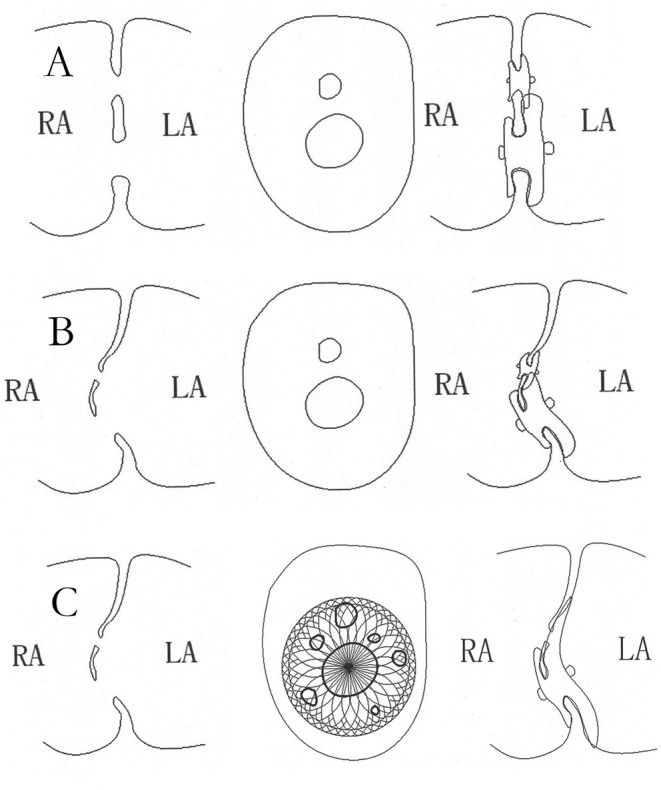
The schematic diagram of closure with standard ASDO in multiple ASDs. The double device in multiple ASDs **(A)** or with ASA **(B)**, the schematic diagram of closure with small-waist–big-edge ASDO in multiple ASDs with ASA **(C)**. ASD, atrial septal defect; ASDO, atrial septal defect occluder; ASA, atrial septal aneurysm.

Four types of occluders were used in the patients, three of which were one of two major categories of the standard ASDO, including Amplatzer Septal Occluder (AGA Medical Corp., Golden Valley, MN, USA), the Chinese domestic-made MemoPart ASDO (SHSMA Corporation & Lepu Medical Technology, Beijing, China), and the Ceraflex ASDO (Lifetech Scientific, Shenzhen, China). The fourth was the small-waist–big-edge ASDO (SHSMA Corporation & Lepu Medical Technology, Beijing, China), which is composed of a connecting waist and large asymmetric discs to cover a wide range of defects, avoiding the use of multiple devices. The difference between standard ASDO and small-waist–big-edge ASDO was shown in Figures 2, 3. The occluder specification is shown in the supplement.

### Transcatheter Closure Procedure

All patients were given local or general anesthesia and intravenous unfractionated heparin. The right femoral vein was cannulated using a Judkins Right catheter (Cook Medical, Bloomington, IN, USA). After setting it in the pulmonary artery and measuring the mean pulmonary artery pressure, the cannula was turned to the left upper pulmonary vein through the ASD, allowing the extra-stiff guidewire to exchange. TTE monitoring was used to ensure the proper distribution, forms, distances from valves to defects, and valvular regurgitation. The sizes of the defects were measured by TTE, including the width of the blood flow. We choose the appropriate devices based on the defect's location and the distances of the defects from each other. The device was delivered to ensure it met the criteria necessary to correct the defect. The occluder was released and guided, using TTE and fluoroscopic detection, through the arteria femoralis–left atrium–ASD–right atrium–femoral vein pathway. If multiple occluders were released, implanting the smaller defect was the priority, and a suitable occluder was chosen according to the jet width of any RS. Successful closure was determined as the absence of any significant complication, such as complete closure or RS jet width of <2 mm, severe arrhythmia, or valve lesion.

### Follow-Up

Patients took aspirin of 3–5 mg/kg/d for 6 months after the operation to prevent thrombosis. All patients were evaluated using an electrocardiogram, x-ray, and TTE 1 day after the operation. Follow-ups were scheduled for 1, 3, 6, 12, and 24 months and every 2 years later, after the operation. At each visit, TTE and electrocardiogram were performed, and x-rays were performed for up to 6 months. RS was defined by the left-to-right shunt across the atrial septum, as shown by the color Doppler. It was graded by the jet width: ≤1 mm is trivial, 1–2 mm is small, 2–4 mm is moderate, and ≥4 mm is large (6–8).

### Statistical Analysis

Data were presented as mean ± SD or median (minimum–maximum) for continuous variables and proportions for categorical variables. The χ^2^-test was used to assess the difference between proportions. Statistical analyses were performed using SPSS version 23.0 (SPSS Inc, Chicago, IL, USA). A two-tailed *p* < 0.05 was considered significant for all analyses.

## Results

### Patient Information

A total of 50 patients (16 males and 34 females) were screened between May 2011 and July 2019. The median age was 5 years (range, 2.3–68 years); the median weight was 20 kg (range, 11.5–60 kg). Six patients had pulmonary hypertension (PH); a patient had moderate PH, and five patients had mild PH. Two patients had incomplete right bundle-branch block. Except for the patients with PH, other baseline characteristics had no significant difference among the five groups. The details are shown in Table 1.

**Table 1:** The patients' demographic characteristic information.

	**Group A (*n* = 16)**	**Group B (*n* = 8)**	**Group C (*n* = 7)**	**Group D (*n =* 7)**	**Group E (*n =* 12)**	***P***
**General characteristic**						
Age (years)	10.86 ± 14.37	10.99 ± 14.64	8.34 ± 9.35	10.37 ± 13.94	11.52 ± 16.84	0.039
Gender (F/M)	12/3	6/5	6/2	8/5	4/2	
Weight (kg)	26.75 ± 16.25	26.62 ± 16.37	24.61 ± 15.09	26.52 ± 16.25	26.54 ± 15.63	0.195
Pulmonary hypertension	6	8	1	5	1	
Complication						
IRBBB				1	1	
Defect						
No. of holes						
2	14	8	6	7		
3	2		1		6	
>3					6	
Diam of the maximum ASD (mm)	7.49 ± 4.67	7.99 ± 5.34	7.42 ± 4.69	7.84 ± 4.58	7.32 ± 4.62	0.02
Diam of ASA (mm)	—	—	—	12.07 ± 6.94	18.24 ± 5.37	0.891
**Device**						
Single standard	14	1	2	2	7	
Double standard		1	4	1		
Three standard			1			
Thin-waist–big-edge	2	6		4	5	
Time of procedure (minutes)	58.33 ± 16.41	66.52 ± 23.24	68.13 ± 24.68	65.90 ± 24.69	64.30 ± 25.99	0.027
Postoperative hospital stay (days)	6.78 ± 0.42	7.59 ± 2.81	7.16 ± 1.50	8.00 ± 3.28	8.30 ± 3.73	0.64
**Follow-up**						
Follow-up (months)	32.33 ± 13.14	26.22 ± 17.97	27.38 ± 15.94	13.82 ± 20.33	24.60 ± 20.43	0.403
PH at the last follow-up time	0	2	0	0	0	
**Complication**						
RS	3	1	3	1	4	
AVB		1			1	

*F, female; M, male; IRBBB, Incomplete right bundle branch block; no. of the holes, the number of defects; Diam, diameter; ASD, atrial septal defect; IDD, interdefect distance; ASA, atrial septal aneurysm*.

### ASD and Procedural Characteristics

As for the defect characteristics, 31 patients had a defect without ASA, 29 patients had double ASDs, and three had three ASDs. The mean diameter of the maximum defect was 7.49 ± 4.67 mm (range, 2.5–26 mm), the mean of IDD was 5.98 ± 3.75 mm (range, 2–16 mm), and 15 patients had a defect with IDD >7 mm. For ASDs with ASA, among the 19 patients in total, seven patients had two defects, six patients had three defects, and six patients had multi–F-ASDs. The mean diameter of the aneurysmal base was 18.27 ± 9.68 mm (range, 10–29 mm). As for the devices used, 26 patients were implanted with a single standard ASDO, six patients were implanted with double standard ASDOs, one patient was implanted with three standard ASDOs, and 17 patients were implanted with a small-waist–big-edge ASDO. The mean of all occluder sizes was 18.00 ± 8.00 mm (range, 6–40 mm); the mean size of standard occluders was 13.00 ± 8.00 mm (range, 6–26 mm), the mean size of small-waist–big-edge occluders was 19.00 ± 8.00 mm (range, 8–26 mm), and there was no statistical significance between the two groups.

**Figure 4:**
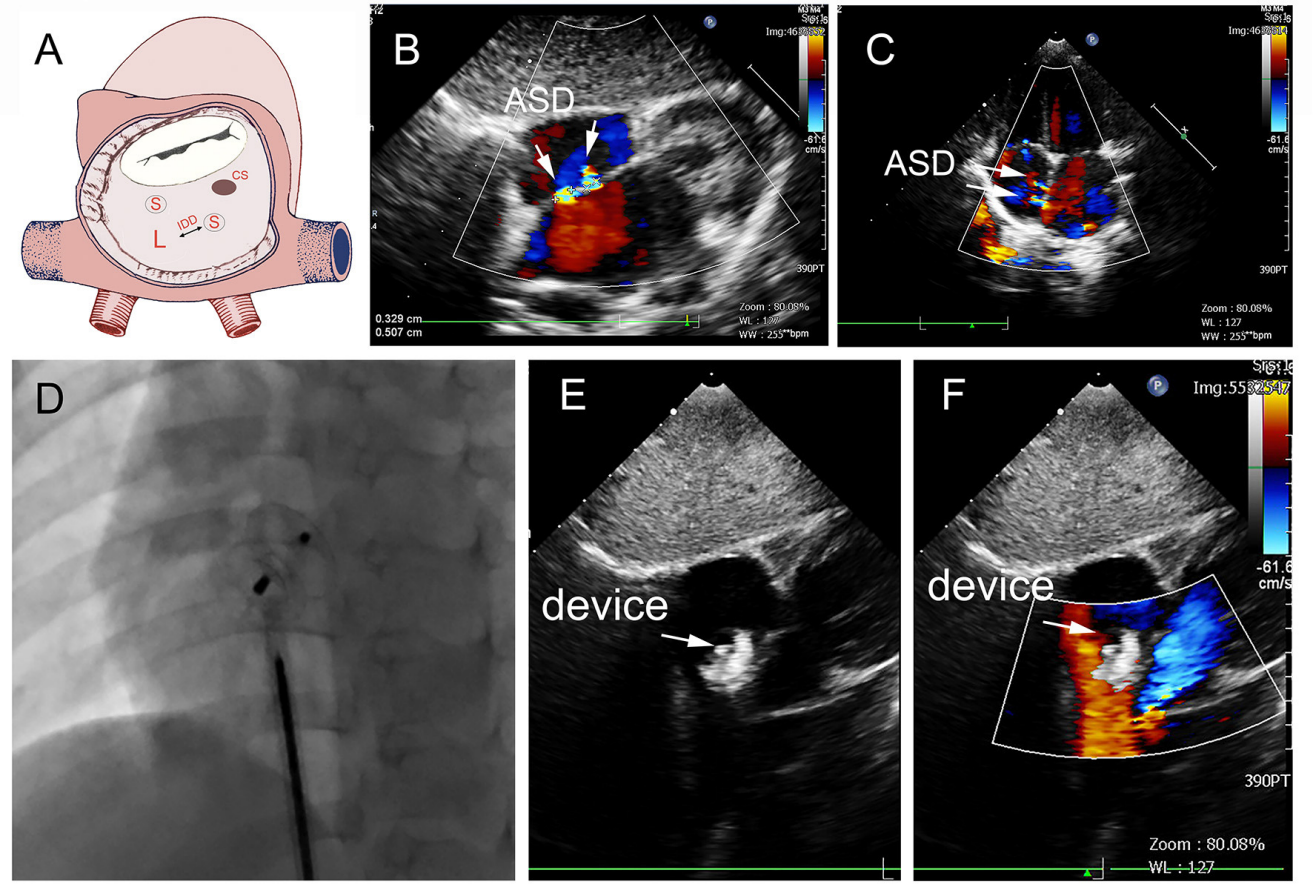
Group A a 29-month-old boy had double adjacent defects, TTE showed the defects were 4 mm and 1.8 mm, the distance of the defects was 3 mm **(A–C)**. In the operation, the 10mm MemoPart ASDO implanted the larger defect **(D)**, and no RS **(E,F)** and any complication happened from immediate postoperation to last follow-up. ASD, atrial septal defect; IDD, inter-defects distance; CS, coronary sinus; S, small ASD; L, large ASD.

In group A, a single standard occluder was used (14/16), except that two patients were implanted with a single small-waist–big-edge ASDO. Typical cases are shown in Figure 4.

**Figure 5:**
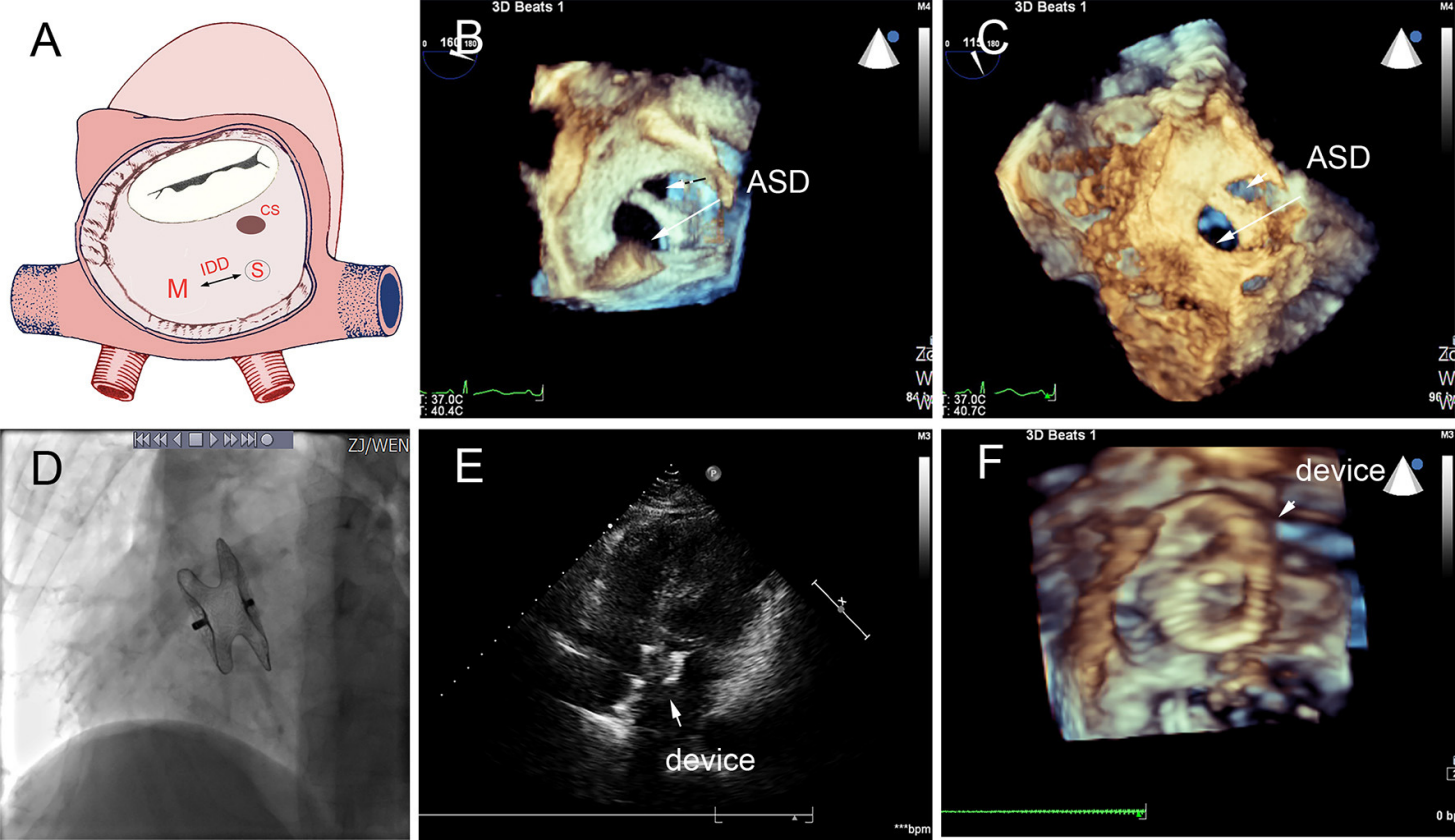
Group B a 68-year-old female, 3D TEE showed the defects were 4 mm and 1.8 mm, the distance of the defects was 3 mm **(A–C)**. In the operation, the 10mm MemoPart ASDO implanted the larger defect **(D)**, no residual shunt **(E,F)** and any complication happened from immediate postoperation to last follow-up. ASD, atrial septal defect; IDD, inter-defects distance; CS, coronary sinus; S, small ASD; M, moderate ASD.

In group B, a single small-waist–big-edge ASDO was used in six patients who had a double defect with IDD of more than 7 mm and two patients who had a soft rim in double defects, which could be covered by a single standard occluder. Typical cases are shown in Figure 5.

**Figure 6:**
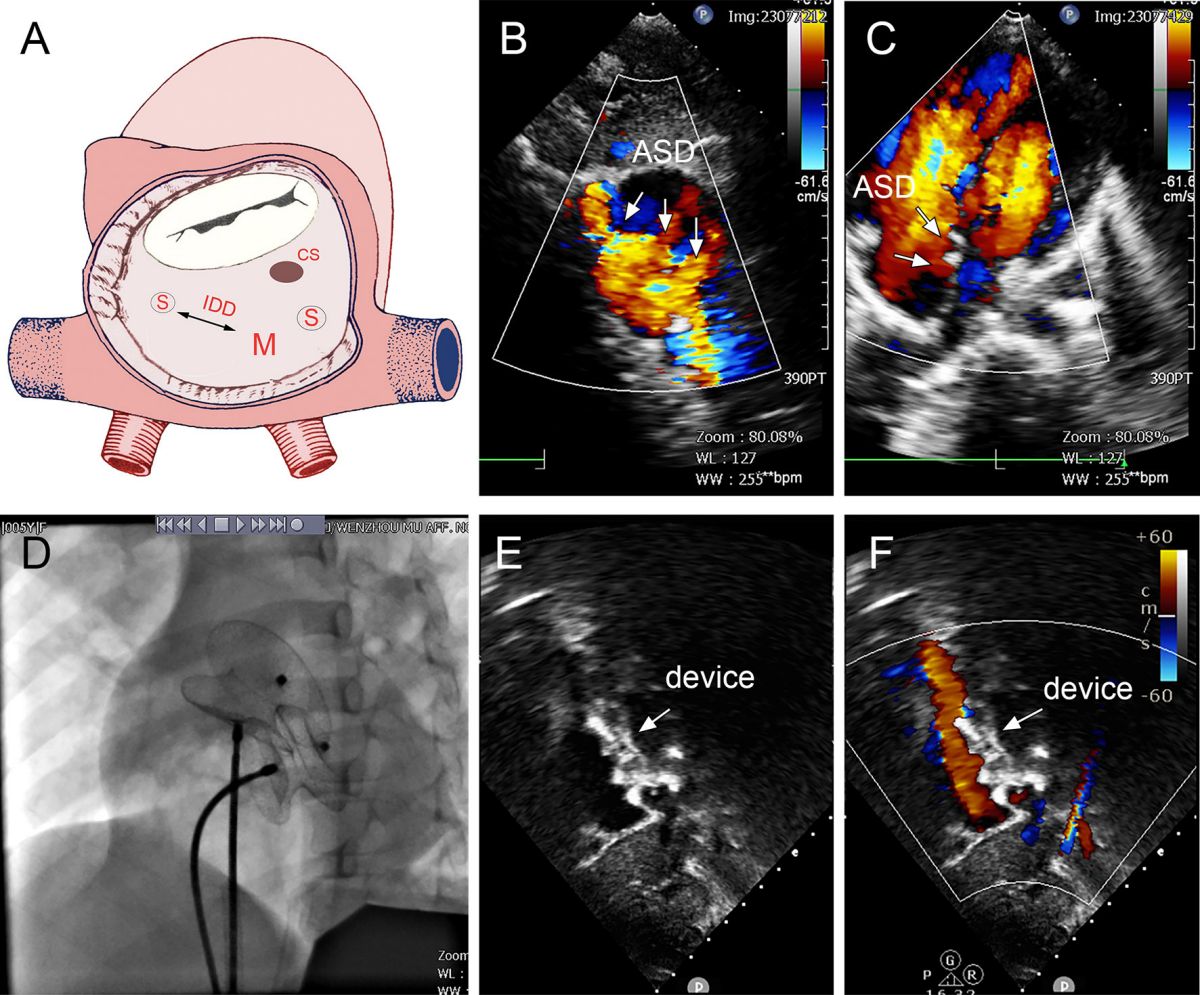
Group C: a 5-year-old girl; the TTE showed three defects (15, 2, and 5 mm); the distance between the largest and smallest hole was 14 mm **(A–C)**. The 16- and 22-mm MemoPart ASDOs were implanted **(D)**, and no residual shunt **(E,F)** and any complication happened from immediate postoperation last follow-up. ASD, atrial septal defect; IDD, interdefect distance; CS, coronary sinus; S, small ASD; M, moderate ASD.

In group C, three AGA ASDOs (12, 12, and 13 mm) were used in six patients each, double occluders in four patients, and a single occluder in two patients; a patient with double ASDOs is shown in Figure 6. One patient had three occluders implanted with no complications seen at the last follow-up. The preoperative TTE showed three defects on the atrial septum, and the patient's father requested transcatheter closure. We released the double occluder remaining moderate RS, whereas five holes were found during the operation. After her father's urgent request, we chose to use one more occluder to attain complete closure. Fortunately, the patient did not experience any complications or adverse events, and the abnormal cardiac structure gradually recovered until the last follow-up. Two patients who had a double defect with long IDD were implanted with a single occluder. We selectively implanted the larger defect by using a larger occluder as much as possible to squeeze the small defect, resulting in a remaining RS below a small degree.

**Figure 7:**
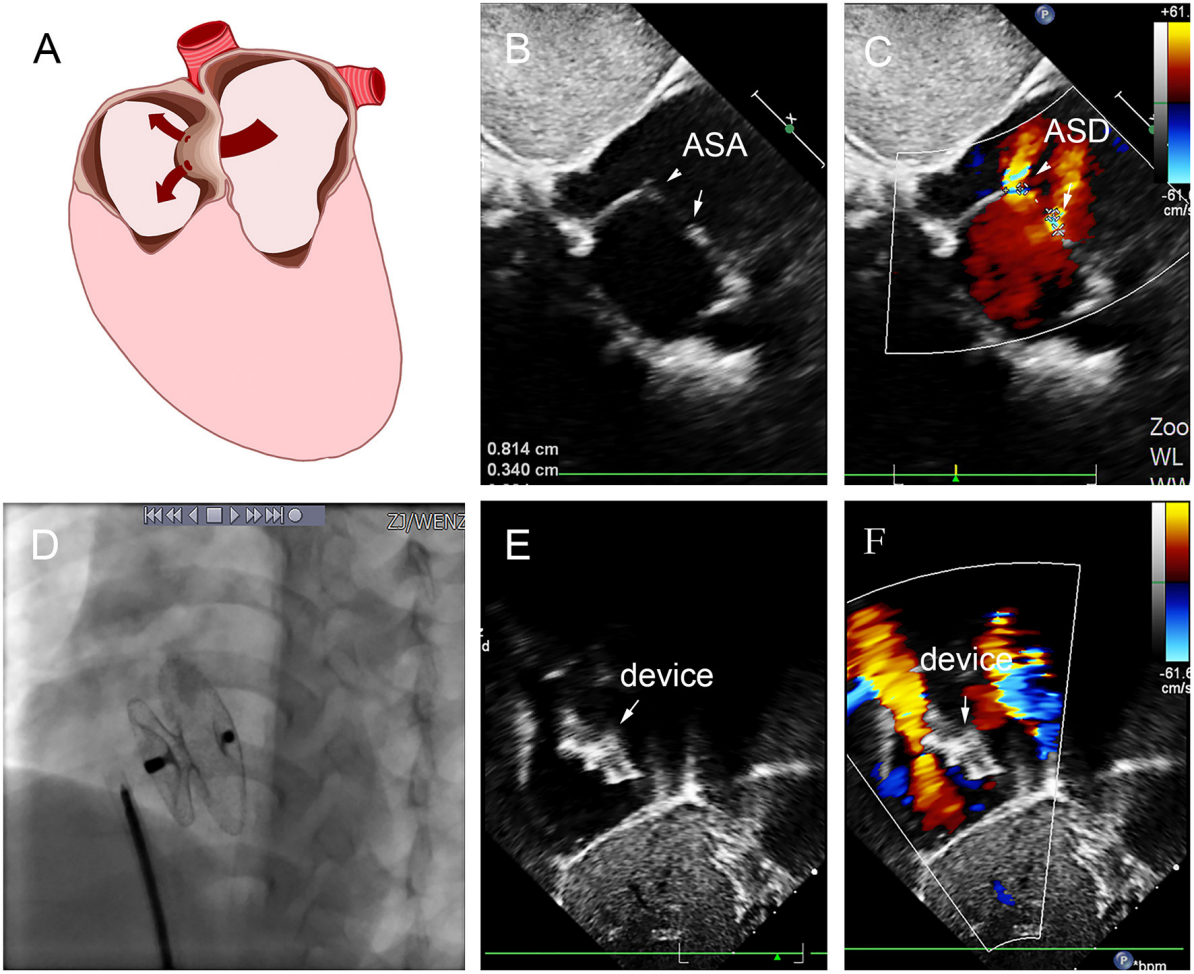
Group D: a 2-year-old girl had double defect **(A)**; the diameters of defects were 2.8 and 3.4 mm, and the diameter of aneurysm base was 10 mm **(B,C)**. We chose an 8 mm small-waist–big-edge ASDO **(D)** had no residual shunt **(E,F)** and any complication at the immediate and last follow-up. ASD, atrial septal defect; ASA, atrial septal aneurysm.

In group D, small-waist–big-edge ASDO was the most used device (4/7), especially when IDD was >7 mm. Two patients were implanted with a single standard occluder, and one patient was implanted with a double standard occluder. A case is shown in Figure 7.

**Figure 8:**
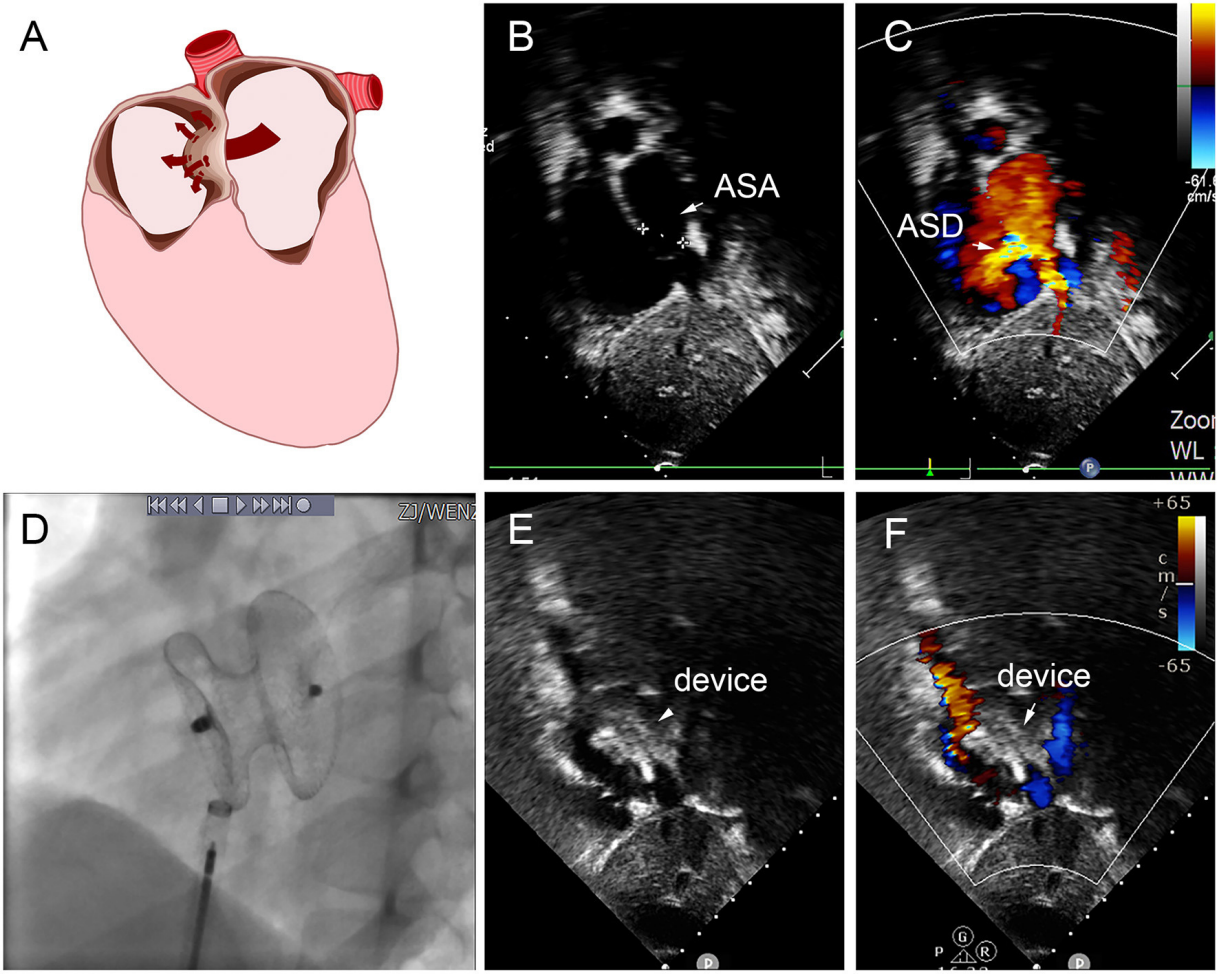
Group E a 8-year-old boy, TTE showed the diameter of aneurysm base was 12 mm, with three defects in the aneurysm (8.8 mm, 2.5 mm, 2.4 mm) **(A–C)**. In the operation, 22 mm MemoPart ASDO implanted the larger defect **(D)**, and no residual shunt **(E,F)** and any complication happened from immediate postoperation to last follow-up. ASD, atrial septal defect; ASA, atrial septal aneurysm.

In group E, five patients were implanted with small-waist–big-edge ASDO. Seven patients with a standard occluder had a large defect and smaller diameter of aneurysm base, a case is shown in Figure 8. The mean diameter of the maximum ASD was 7.43 ± 2.60 mm, which was larger than the patients with small-waist–big-edge (6.83 ± 4.40 mm). The aneurysm base diameter was smaller than that of the patients with small-waist–big-edge (9.52 ± 5.38 vs. 18.27 ± 5.34 mm, *P* < 0.05).

The time for performing the procedure, postoperative hospital stays, and follow-up time had no significant differences among the five groups.

### Follow-Up Results

The mean follow-up time was 25.76 ± 22.53 months (range, 6–72 months). Besides RS and atrioventricular block (AVB), no other complications were found in any of the patients. The immediate complete closure rate was 38 of 50 (76%), and at the 6-month follow-up, the complete closure rate was 92%, only one patient in groups A, C, D, and E, respectively. The information is shown in Table 2.

**Table 2:** Residual shunt after transcatheter closure.

**Groups**		**3 days**	**1 month**	**3 months**	**6 months**	**12 months**	**24 months**	**36 months**	**>60 months**
Total	*n =* 50	12 (24%)	10 (20%)	6 (12%)	4 (8%)	4 (8%)	3 (6%)	2 (4%)	N
Group A	Standard (*n =* 14)	3 (6%)	1 (2%)	1 (2%)	1 (2%)	1 (2%)	N	N	N
	Thin-waist–big-edge (*n =* 2)	0	0	0	0	0	0	N	N
Group B	Standard (*n =* 2)	1 (2%)	1 (2%)	1 (2%)	0	0	0	0	0
	Thin-waist–big-edge (*n =* 6)	0	0	0	0	0	0	N	N
Group C	Standard (*n =* 7)	3 (6%)	2 (4%)	1 (2%)	1 (2%)	1 (2%)	1 (2%)	N	N
	Thin-waist–big-edge (*n =* 0)	0	0	0	0	0	0	N	N
Group D	Standard (*n =* 3)	1 (2%)	1 (2%)	1 (2%)	1 (2%)	1 (2%)	1 (2%)	1 (2%)	N
	Thin-waist–big-edge (*n =* 4)	0	1 (2%)	1 (2%)	1 (2%)	1 (2%)	1 (2%)	1 (2%)	N
Group E	Standard (*n =* 7)	1 (2%)	1 (2%)	1 (2%)	0	0	0	N	N
	Thin-waist–big-edge (*n =* 5)	3 (6%)	3 (6%)	0	0	0	0	N	N

*ASDO, atrial septal defect; N, no follow-up time was reached*.

**Figure 9:**
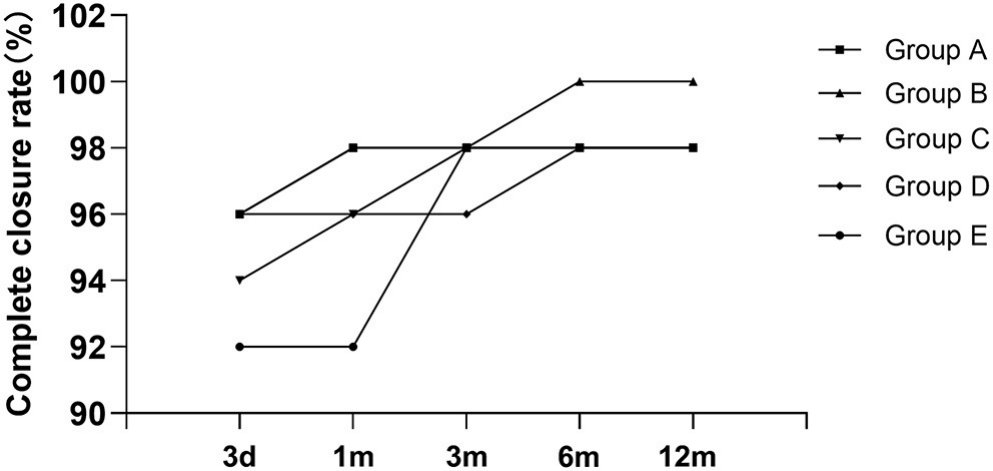
The condition of residual shunt after transcatheter closure. d, days; m, month(s).

**Figure 10:**
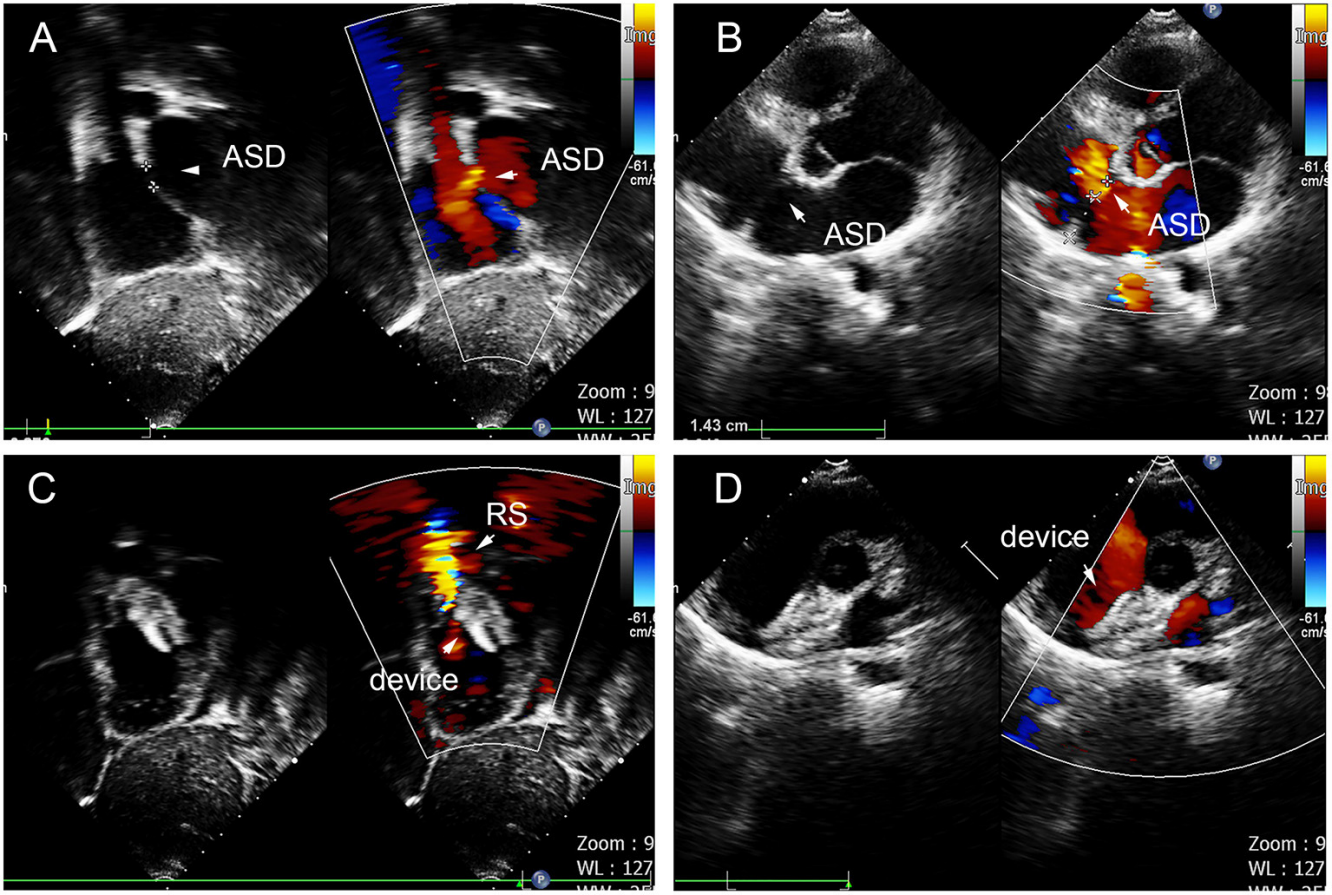
Double defects in the atrial septum **(A,B)**, and there remained residual shunt at last follow-up **(C,D)**. ASD, atrial septal defect; RS, residual shunt.

**Figure 11:**
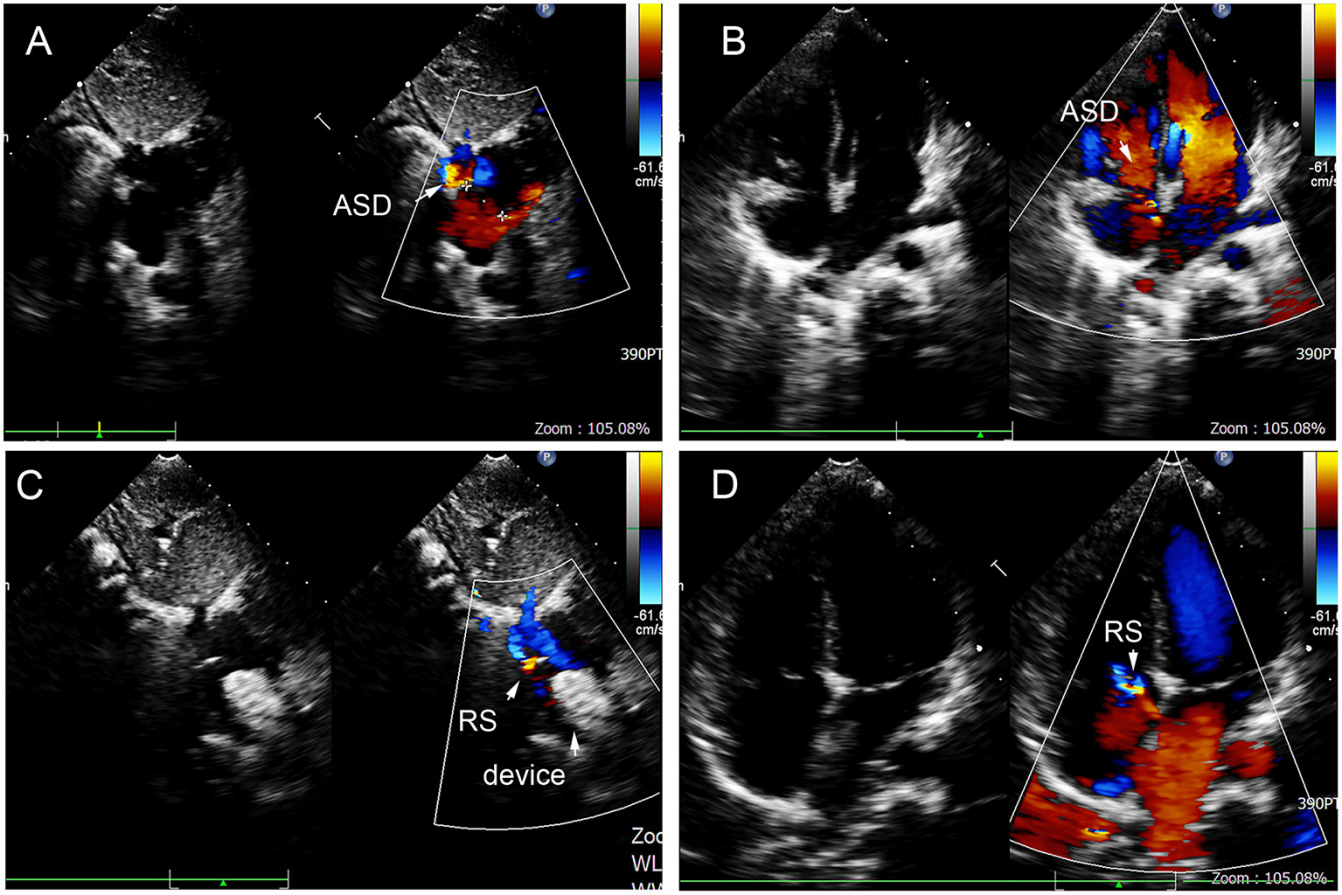
Smaller defect near inferior vena cava double defects in the atrial septum **(A,B)**, and there remained residual shunt near inferior vena cava **(C,D)**. ASD, atrial septal defect; RS, residual shunt.

**Figure 12:**
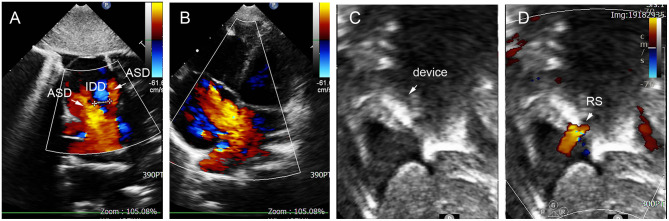
ASA in 3-year-old girl had 18 mm aneurysm base, and double defects on the aneurysm with the IDD = 9 mm **(A,B)**, remaining small residual shunt at 36-month follow-up **(C,D)**. ASD, atrial septal defect; IDD, inter-defects distance; RS, residual shunt.

**Figure 13:**
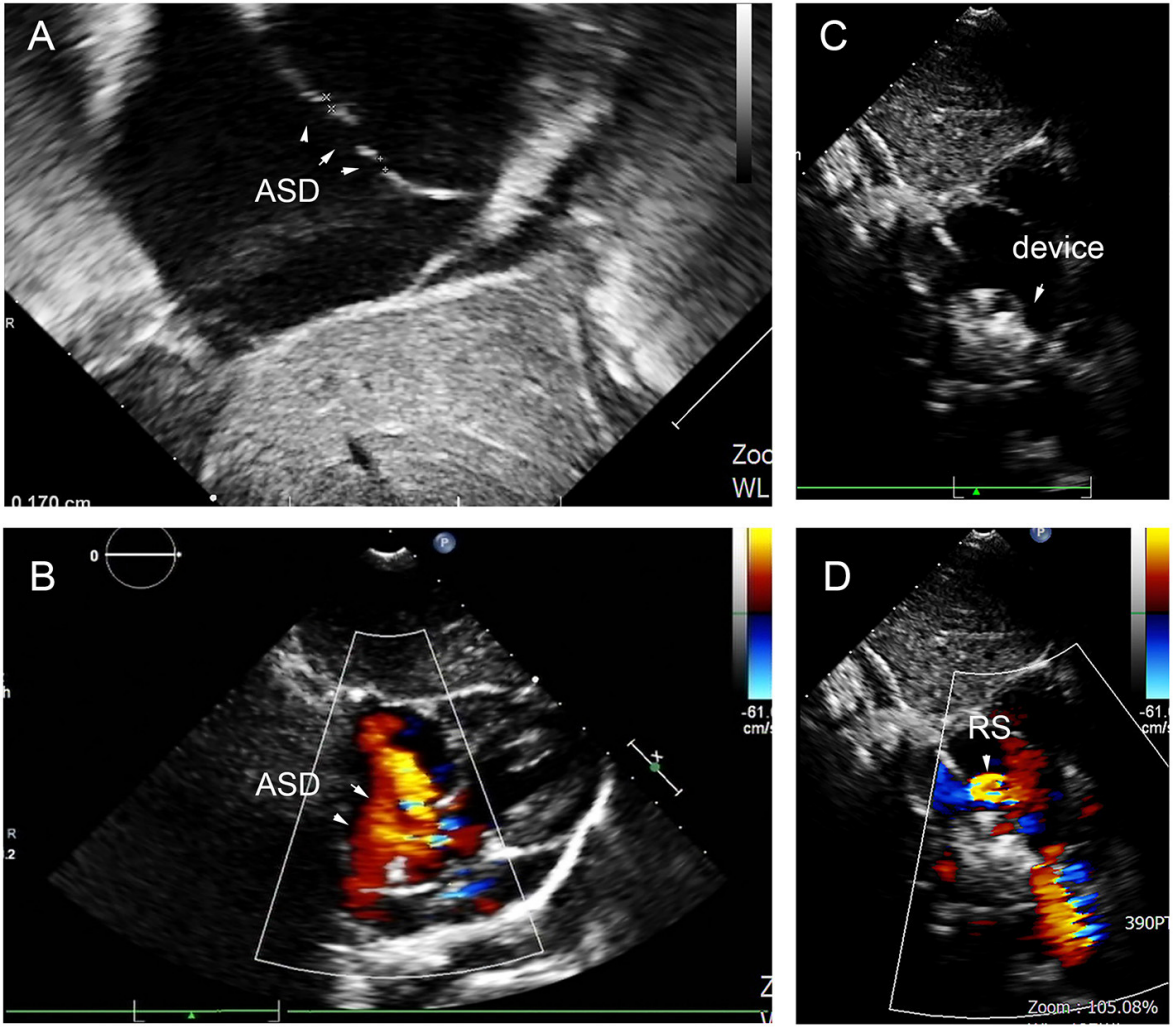
Multiple trivial defects in the atrial septum **(A,B)**, and there remained small residual shunt at 48-month follow-up **(C,D)**. ASD, atrial septal defect; RS, residual shunt.

At the last follow-up, four patients had a small RS in A, C, D, and E. The details are shown in Figure 9. A 6-year-old girl in group A who had double ASDs with 2-mm IDD was implanted with a 16-mm MemoPart ASDO and had a small RS until the 12-month follow-up, and the details are shown in Figure 10. In group B, an 8-year-old girl also had double ASDs (5 and 1.7 mm) with 23-mm IDD, with the smaller defect near inferior vena cava (IVC). A 12-mm MemoPart ASDO was implanted in the larger one, and she had a remaining small RS at the 24-month follow-up; the details are shown in Figure 11. One patient with a 22-mm MemoPart ASDO had immediate trivial RS in group D; she had an 18-mm aneurysm base, and the maximum defect was 12 mm. The occluder was implanted in the larger one, but she had a remaining small RS at the 36-month follow-up; the details of RS are shown in Figure 12. One patient had multiple trivial defects in the atrial septum in group E, with the maximum defect being 4 mm, and a small defect was 9 mm from the central defect. An 8-mm small-waist–big-edge ASDO was implanted, but there was a remaining small RS at the 48-month follow-up; the details of RS are shown in Figure 13; however, she had no repeated respiratory tract or cardiac structural changes through the last follow-up.

After a short- to midterm follow-up, two patients had an AVB. One patient had a continuous, prolonged PR interval, from 125 to 216 ms, but recovered to normal with hormonotherapy for 1 month. Another patient with a 24-mm device had three-degree AVB. After adrenocorticosteroid therapy, no abnormal electrocardiograph was found at the 6-month follow-up. The details are shown in Table 3. A 2-year-old-girl implanted with an 8-mm device had a prolonged PR interval, from 125 to 215 ms after the operation. We administered a 4-day regimen of low-dose hormone therapy to reduce edema, which returned to normal after 1 month. Another patient implanted with a 10-mm device had a third-degree AVB 1 day after the operation. We used large-dose hormone therapy for 3 weeks, and electrocardiogram recovered to a first-degree AVB, and the Holter monitor did not indicate a supra-II AVB. At the 6-month follow-up, the PR interval was 172 ms (heart rate = 81 beats/min).

**Table 3:** The patients with AVB characteristics.

**General data**				**Defect**		**pre-operation**		**Closure**	**Postoperation**		**Follow-up**			
**No**.	**Sex**	**Age (year)**	**Weight (kg)**	**#**	**Size (mm)**	**PR (ms)**	**HR (bpm)**	**Size (mm)**	**PR (ms)**	**HR (bpm)**	**Degree**	**Time (months)**	**PR (ms)**	**HR (bpm)**
14	F	2.5	11.5	2	3.4	126	97	8	215	104	I	6	138	81
16	F	5.5	17	M	2	150	118	10	134	124	III	6	172	81

*AVB, atrioventricular block; #, the number of defects; HR, heart rate; bpm, beats per minute; F, female; M, multiple*.

## Discussion

An ASD is a common congenital heart disease. Multiple ASDs comprise 9.7–17.9% of ASDs (3, 9–11). A total of 50 patients were treated with percutaneous transcatheter closure for multiple ASDs in our hospital between March 2011 and July 2019. Multiple ASD is difficult to treat with percutaneous catheter closure, because it often has ASA, a deficient rim, or a long IDD (IDD >7 mm). Masseli et al. reported that 148 multiple ASDs patients achieved successful closure, and 87% had complete closure (11). Numan summarized 13 patients implanted with a cribriform ASD device, achieving a successful and safe result. Most of the patients (69%) had an aneurysm, and the researchers speculated that the cribriform device was a suitable device for treatment in the presence of an aneurysm (12). Because of the different characteristics of multiple ASDs and the importance of selecting a suitable occluder or diverse closure strategy to perform a successful percutaneous closure, we share our single-center individualized experience with percutaneous transcatheter closure involving 50 multiple ASD patients.

We divided the patients into five groups by their IDD and ASA. An ASA's presence increases the incidence of embolism and arrhythmia, so it is important to complete closure of the aneurysm undergoing transcatheter closure for ASD as soon as possible (13, 14). However, for covering the F-ASDs, the standard ASDO had an oversize waist and would damage the aneurysm, so it was important to choose a suitable occluder. Because of a standard occluder's structure, for double ASDs with more than 7-mm IDD, the use of multiple devices was recommended. Also, the small-waist–big-edge ASDO was a new option to cover long IDD defects.

Double ASDs with bigger and smaller holes could be completely closed by implanting the bigger hole and covering the smaller ones. Szkutnik et al. reported that 41 of 363 patients undergoing transcatheter closure had double ASDs, with a distance between the defects of ≤7 mm and that the use of single device for double defects had a similar closure rate to the use of a standard ASD (15). The report also found that if the IDD was more than 7 mm or the implanted defect had a deficient rim, RS or valve damage would arise. The thin-waist–big-edge ASDO has a 6–24-mm size (measured at the waist) in 12-mm increments for the left disc. The benefit of its structure is that the occluder can cover a broader range of 12 mm and completely close defects with an IDD of more than 7 mm. When a deficient rim is present, the thin-waist–big-edge ASDO can be implanted when there is a sufficient rim defect (the smaller defect) and cover the deficient rim. However, because the maximum size is limited to 24 mm, resulting in a restricted large left disc, careful consideration should be used in selecting the thin-waist–big-edge ASDO if the implanted defect is large. In our study, one patient, for example, had a double defect, with the larger being 18 mm and the smaller defect having a deficient rim (IVC), which was 7 mm from the implanted defect. We selected the 24-mm thin-waist–big-edge ASDO for the first attempt, but still had moderate RS after implantation.

Nevertheless, we achieved complete closure after using 32- and 16-mm standard occluders. Therefore, selecting the appropriate device for transcatheter closure for double ASDs required assessing the diameter of the defects (especially the implanted defect), the rim of the implanted defect, the distance from the implanted defect to the adjacent hole, and assessment of the vital cardiac structures. We focused on any aneurysm morphological features, the number of the holes, and the surrounding tissue rims using TEE or TTE.

ASA is a rare congenital anomaly, with an incidence being 2–3%, and may be associated with multiple septal fenestrations (13, 14). For a successful transcatheter closure, ASDs should have adequate rims, but ASAs may not have adequate support for implanting an occluder and may even be torn by a large occluder. Fatma et al. proposed four classifications of ASA patients suitable for device closure (16), based on the success of transcatheter device placement. So, we roughly divided our patients into two groups according to the number of holes. If the ASA had adjacent double defects, and the maximum ASD size was moderate (5–15 mm) or larger (>15 mm) ASD, the standard occluder was recommended. If the distance between the double defects was long or the defects were small or had a trivial shunt, the thin-waist–big-edge ASDO was appropriate. However, if there was a long distance between defects (IDD >12 mm), the use of double occluders was an alternative treatment for a successful closure. In the case of aneurysms with multiple holes, a cribriform device was the first choice. Numan et al. showed that eight of the 16 reported patients had multi–F-ASDs, and 92% of these patients had complete closure after 1 year (12). The thin-waist–big-edge ASDO has a similar structure to cribriform devices. If a patient has a small hole on the atrial septum and a large aneurysm base, the thin-waist–big-edge ASDO could cover multiple holes with 12-mm increments left disc. Its thin waist can match a small defect and reduce the squeezing of the aneurysm. If the central defect was a medium or large defect, a single standard occluder could completely close the aneurysm.

Of our 50 cases, four patients had RS immediately after surgery, with only one patient still having RS after the last follow-up, providing a closure rate of 98%. Farhaj et al. reported on 150 multiple ASDs patients who underwent transcatheter closure, with 58 (38.7%) of the patients having immediate RS, and the complete closure rate was 80% at the 12-month follow-up (2). Even though RS was inevitable, Veldtman GR report that if the width of RS<3 mm, cardiac function recovered, murmur disappeared, the heart structure could be recovered, it is unnecessary for secondary closure (17). In our study, two patients with ten-mm IDD defects had immediate trivial RS (jet width<1 mm), could not be closed by a standard occlude but were gradually able to achieve complete closure at 3 months. We speculated that the trivial RS (jet width<1 mm) could be closed with the benefit of endothelialization.

After mid- to long-term follow-up, AVB was a significant complication in two patients. The atrioventricular node was located deep in the Koch's triangle's right atrium, emitting the bundle (18). Implanting of the occluder would only squeeze the atrial septum, also causing edema because of operation procedures stimulus. It is relevant to the compression and edema at Koch's triangle and causes transient dysfunction of the atrioventricular node. The occurrence of AVB was related to the compression and abrasion caused by the occluder (19). Device size >19 mm or indexed size >0.18 mm/cm was the major predisposing factor for AVB (20). Li et al. showed the pathology of atrioventricular node edema in AVB patients after the operation and believed that corticosteroids could relieve inflammatory edema and benefit AVB recovery (18). Our study reports two patients with the small-waist–big-edge ASDO who had transient AVB within 3 days after closure and regained normal conduction velocity after medication. We speculate that AVB may be related to atrial septum inflammatory edema, and corticosteroids medication is an effective treatment.

### Limitations

This article describes a retrospective study limited by the small sample; more multiple-ASD patients are required to better determine the transcatheter closure experience. Second, theoretically, small-waist–big-edge ASDO can cover a defect with a 12-mm IDD, but in fact the longest IDD in our successful cases is 11 mm. Third, although one child suffered from serious AVB and benefited from medication, the complication related to the device disc's diameter became more important, especially the small-waist–big-edge ASDO. Finally, long-term complications need to be observed through a longer follow-up. Cardiac abrasion generally occurs within 1 month after closure, but can appear 3 years after closure (21). Multiple ASDs could achieve complete closure using a single device instead of multiple standard devices, but more cases and longer follow-ups are needed to determine this.

## Conclusions

Depending on the different characteristics of multiple ASDs, individualized experience with percutaneous transcatheter closure for multiple ASDs was effectively achieved in a single-center study. After mid- to long-term follow-up, the multiple ASDO and small-waist–big-edge ASDO had no major adverse events or complications.

## Data Availability Statement

The raw data supporting the conclusions of this article will be made available by the authors, without undue reservation.

## Ethics Statement

The studies involving human participants were reviewed and approved by the Ethics Committee of the Second Affiliated Hospital of Wenzhou Medical University. Written informed consent to participate in this study was provided by the participants' legal guardian/next of kin.

## Author Contributions

RW conceived the study. YZ wrote the manuscript. ZW analyzed the results. JJ, TW, SZ, HQ, and QW collected the clinical data. All authors contributed to the article and approved the submitted version.

## Conflict of Interest

The authors declare that the research was conducted in the absence of any commercial or financial relationships that could be construed as a potential conflict of interest.
